# Genome-wide association study of rheumatoid arthritis by a score test based on wavelet transformation

**DOI:** 10.1186/1753-6561-3-s7-s8

**Published:** 2009-12-15

**Authors:** Renfang Jiang, Jianping Dong, Yilin Dai

**Affiliations:** 1 Department of Mathematical Sciences, Michigan Technological University, 1400 Townsend Drive, Houghton, MI 49931, USA

## Abstract

**Background:**

We have conducted a genome-wide association study on the Genetic Analysis Workshop (GAW) 16 rheumatoid arthritis data using a multilocus score test based on wavelet transform proposed recently by the authors. The wavelet-based test automatically adjusts for the amount of noise suppressed from the data. The power of the test is also increased by using the genetic information contained in the spatial ordering of single-nucleotide polymorphisms on a chromosome.

**Results:**

After adjusting for the effect of population stratification, the test identified some previously discovered rheumatoid arthritis susceptibility loci (*HLA-DRB1 *and rs3761847) as well as some loci (rs2076530 and rs3130340) known to have association with sarcoidosis and bone mineral density. It was previously reported that patients with rheumatoid arthritis have elevated prevalence of sarcoidosis and have reduced bone mass.

**Conclusion:**

This new test provides a useful tool in genome-wide association studies.

## Background

In genome-wide association studies, the first choice of tests is usually a single-marker test. If a single-nucleotide polymorphism (SNP) has a strong association with a disease, single-marker tests should have higher power than multilocus tests. Multilocus tests can achieve higher power if several SNPs are associated with the disease. However, the potential high power of multilocus tests could be diminished as an increased number of markers results in an increased number of degrees of freedom. Therefore, reducing the number of degrees of freedom is essential to increasing the power of multilocus tests. Different strategies were introduced to reduce the number of degrees of freedom. Tests based on haplotype sharing (the longest continuous interval of matching alleles between haplotypes) effectively reduce the number of degrees of freedom [1,2]. Another popular approach is to apply principal components analysis (PCReg) [3]. A score test based on the Fourier transform [4] is another attempt to reduce the degree of freedom, thereby increasing power. Instead of using the genotype data, weighted Fourier transform coefficients of the genotype data are used to form a score test.

Many multilocus association tests are not affected by permuting spatial order of SNPs; thus, they do not use the information contained in the ordering of SNPs. For example, the results of logistic regression will not change if the order of SNPs are permuted. The same is true for the test obtained by fitting a regression function with one SNP followed by Bonferroni correction to find the global *p*-value. PCReg is also invariant under the permutation of SNPs. We provide a proof of the above claims as follows (See Wang and Abbott [3] for notations in the following proof.). Let *G *be a matrix of coded genotypes and suppose it is centered. The variance-covariance matrix of genotypes is *A *= *G*^*T*^G/(*n *- 1) = *VDV*^-1^, where *D *is a diagonal matrix with eigenvalues of A as diagonal entries, and the columns of *V *are the eigenvectors. The regression model is *y *= *GV*_1_*b *+ ε, where *V*_1 _is the first several columns of *V*. After permutation of SNPs, the columns of *G *are also permuted, and G becomes GP, where P is a permutation matrix. Since (*GP*)^*T *^(*GP*) = (*P*^*T*^*V*) *D*(*P*^*T*^V)^-1^, the eigenvalues are not changed by the permutation, and the matrix of eigenvectors becomes *P*^*T*^V. The regression model  remains unchanged. For the tests based on shared haplotype length, changing the ordering of SNPs could mean a shortened shared length, and the association between the disease and the SNPs could disappear. Therefore, the ordering of SNPs contains important genetic information, and ignoring it could lower the power of association tests.

We recently proposed a score test based on wavelet transform [5,6], which is used in this report. This test has three advantages. First, it uses the wavelet transformation of genotype data instead of the genotype data. The wavelet transform is designed to handle unsmooth noisy signals. Genetic data are usually unsmooth and can be dealt with by the wavelet transform naturally. Second, it uses an empirical Bayes thresholding [7]. It was proved by Johnston and Silverman that this thresholding effectively suppresses noise from data [7]. Therefore, it increases the power of the test. The exact amount of noise being reduced depends on the specific problem. Third, because our method views multilocus genotypes of an individual as a discretized function, the spatial ordering of the SNPs on a chromosome is taken into consideration, which increases the power of the test.

## Methods

There is a total of 2,062 individuals consisting of 868 cases and 1,194 controls in the North American Rheumatoid Arthritis Consortium (NARAC) data for Genetic Analysis Workshop (GAW) 16. These individuals were genotyped on the 550 k Illumina SNP chip. We analyzed 22 autosomal chromosomes in this report. SNPs satisfying one of the following criterion were excluded: missing genotype rate >0.05, or minor allele frequency < 0.05, or having *p*-values < 0.00001 in the Hardy-Weinberg equilibrium test. About 12.8% of SNPs were removed. Missing genotypes were imputed by fastPHASE [8]. We adopted a strategy of a moving window of eight SNPs without overlapping (the first window contains SNPs 1-8, the next window contains SNPs 9-16, etc.) on the chromosome. We have tried windows of sizes 16 and 32 SNPs and there was not much difference. Overlapping windows were also tried and again, the results were similar. Computational burden increases when large windows or overlapping windows were used. The *k*^th ^window is from the (8(*k *-1)+ 1)^th ^SNP to the 8*k*^th ^SNPs. Let *G*_*k *_be the corresponding eight columns of the genotype matrix *G*. Different ways of coding SNPs do make a difference. The SNPs in *G*_*k *_were recoded to maximize the number of positive pairwise correlations. This removes any ambiguity in the coding of genotypes. In our simulation studies, the recoding increased the power of the test while keeping the type I error rate in check.

We applied EIGENSTRAT [9] to remove the effects of population stratification. The top three eigenvectors obtained by EIGENSTRAT were used to adjust genotypes and phenotypes as suggested by Price et al. [9]. Let *G *be the genotype matrix, *C *be the matrix of the principal component vectors, and *Y *be the vector of phenotypes. The adjusted genotype matrix is  = *G *- *CC*^*T*^*G *and the adjusted phenotype vector is  = *Y *- *CC*^*T*^*Y*. Before the adjustment, the inflation factor [10]* λ*_GC _= 1.399, which reduced to 1.025 after the adjustment. To avoid a possible bias, all chromosomes except 6 and 8 were included to produce the eigenvectors, which were used to adjust genotypes on chromosomes other than 6. Chromosome 6 was used to produce eigenvectors to adjust genotypes on chromosome 6.

Consider the *k*^th ^window and the corresponding matrix of adjusted genotype . Applying wavelet transform, thresholding, and inverse wavelet transform consecutively on the *i*^th ^row of  resulted in the modified genotype (*x*_*i*1_, *x*_*i*2_, ..., *x*_*im*_) of the *i*^th ^individual. Subtract the mean of the *j*^th ^column of *X *= (*x*_*ij*_) from *x*_*ij *_such that the mean of each column of *X *= 0. Let  = (*y*_1_, *y*_2_, ..., *y*_*n*_) be the adjusted phenotype of *n *individuals. Assume a generalized linear model [11]* f*(*E*(*y*_*i*_)) = *α *+ *X*_*i*_*β *between *X *and *Y*, where *f *is a link function. Let  be the score statistic for the markers. The variance of *U*_*j *_under the null hypothesis can be estimated by  The global score statistic of this window is 

The global *p*-values of the wavelet-based test were obtained as follows. We randomly permuted the phenotypes (case-control status) 5,000 times, and received 5,000 sets of permuted phenotypes. Adjust each set with the principal component vectors, and they are still called permuted phenotypes. At each window of eight SNPs, the value of *T *was calculated 5,001 times, using the adjusted phenotypes and 5,000 sets of permuted phenotypes, respectively. In *M *windows along the chromosomes, we have 5000 *M *values of *T *with permuted phenotypes, denoted by a 5,000 × *M *matrix (*T*_*ij*_), where *T*_*ij *_is the absolute value of *T *at the *j*^th ^window using the *i*^th ^set of permuted phenotypes. Let *m*_*i *_= max_*j *_*T*_*ij *_be the maximum absolute value of *T *at *M *windows using the *i*^th ^set of permuted phenotypes. Let *T*_*j *_be the absolute value of *T *at the *j*^th ^window using the adjusted phenotypes. The global *p*-value of the wavelet-based test at the *j*^th ^window is the proportion of *m*_*i *_> *T*_*j*_: global *p*-value of *T*_*j *_= #{*m*_*i*_|*m*_*i *_> *T*_*j*_}/5,000.

## Results and discussion

After correcting for population stratification, significant signals were only found on chromosomes 6 and 9. Four windows on chromosome 6 attracted our attention. The first window (rs9268005, rs3130340, rs3115553, rs9268132, rs926070, rs6935269, rs7775397, rs17422797) contains rs3130340, which was identified to have association with bone mineral density and fractures [12]. It has been reported that a large proportion of men with rheumatoid arthritis (RA) had reduced bone mass [13]. The *p*-value at this window is 0.0074. The second window (rs4424066, rs12529049, rs3117099, rs3117098, rs3817973, rs1980493, rs2076530, rs4248166) contains rs2076530, which is associated with sarcoidosis [14]. Increased prevalence of sarcoidosis among RA patients has been reported [15]. The association between sarcoidosis, RA, and rs2076530 is an interesting phenomenon. The association between rs2076530 and RA was investigated before, but whether it is a causal SNP for RA or its effect is merely a carryover effect of nearby haplotypes [16] merits further investigation. The third window (rs2395182, rs3129890, rs9268832, rs6903608, rs2395185, rs477515, rs2516049, rs2858870) contains *HLA-DRB1*, which has long been identified as a major genetic risk contributor to RA [17]. The last window (rs9275224, rs5000634, rs6457617, rs2647012, rs9357152, rs10484561, rs9275313, rs1794282) contains rs6457617, which has also been reported as being associated with RA [18]. The *p*-values of the test at the above three windows are less than 0.0002 (it is 0 after 5,000 permutations). The most significant window on chromosome 9 (rs1953126, rs10985073, rs3761847, rs10985095, rs10985097, rs2900180, rs12235400, rs10985112) which contains rs3761847 and it was identified as a risk locus for RA [19], has a *p*-value 0.53.

**Figure 1:**
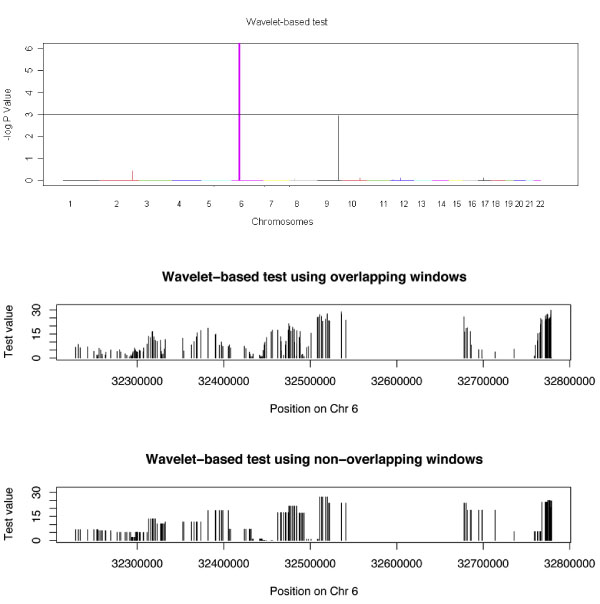
***p*-Values on 22 chromosomes, and comparison of windows**. The plot on the left contains the *p*-values of the wavelet-based test on 22 chromosomes. The horizontal line indicates 5% significance level. The plots on the right are a comparison of overlapping windows (top) and non-overlapping windows (bottom) for a 550-kb region on chromosome 6.

**Figure 2:**
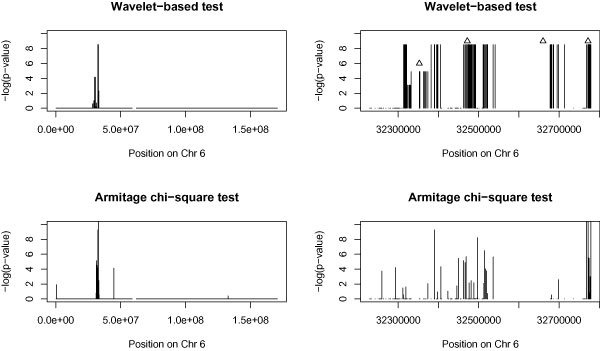
**Comparison with single marker test**. Comparisons of the wavelet-based test and the Armitage χ^2 ^test on chromosome 6. The plots on the left are for the whole chromosome 6, and the plots on the right are fine mapping results for a 550-kb region of chromosome 6. The triangles indicate the positions (from left to right) of rs3130340 (associated with bone mineral density and RA), rs2076530 (associated with sarcoidosis and RA), HLA-DRB1 (Note: no data from 32.54 Mb to 32.67 Mb), and rs6457617 (associated with RA).

We applied the wavelet-based test on a moving window of eight SNPs with overlapping (the first window contains SNPs 1-8, the second window contains SNPs 2-9, etc.) on a 550-kb region of chromosome 6 for fine mapping. The results are shown in Figure 1. We have tried other window sizes (16 and 32) and found similar results (not shown). Comparisons of the wavelet-based test using overlapping and non-overlapping windows showed no significant differences. In Figure 2, we compared the wavelet-based test with Armitage χ^2 ^test. If *p*-value of the wavelet-based test is 0 after 5,000 permutations, we set the *p*-value as 0.0002 in order to calculate log(*p*-value) in Figures 1 and 2, which makes 0.0002 the smallest possible *p*-value in this study. Some significant loci (*HLA-DRB1*, rs2076530, rs6457617) were identified by both tests. However, the Armitage χ^2 ^test was not significant around rs3130340 (*p*-value after Bonferroni correction was 1), while the *p*-value of the wavelet-based test at the window containing the SNP was 0.0074, after correcting for multiple testing.

## Conclusion

A wavelet-based multilocus score test was applied in a genome-wide association study on RA data followed by fine mapping of regions identified in our genome-wide association study. Several statistically significant risk loci for RA were identified after adjustment for population stratification. Some windows contain genes and/or SNPs (*HLA-DRB1*, rs3761847, and rs6457617) previously associated with RA. Some windows contain SNPs (rs2076530, rs3130340) previously associated with sarcoidosis [14] or bone mineral density and fractures [12], all of which have been reported as being related with RA [13,15].

## List of abbreviations used

NARAC: North American Rheumatoid Arthritis Consortium; PCReg: Principal components analysis; RA: Rheumatoid arthritis; SNP: Single-nucleotide polymorphism

## Competing interests

The authors declare that they have no competing interests.

## Authors' contributions

RJ and JD both contributed in development of the statistical test, provided simulation strategies, and drafted the manuscript. RJ also participated and guided the numerical calculations. YD carried out part of the programming work. All authors read and approved the manuscript.
